# Letter to the Editor Regarding First Admission Neutrophil–Lymphocyte Ratio and Ischemic Stroke

**DOI:** 10.5041/RMMJ.10456

**Published:** 2021-10-25

**Authors:** Rujittika Mungmunpuntipantip, Viroj Wiwanitkit

**Affiliations:** 1 Private Academic Consultant, Bangkok, Thailand; 2 Honorary Professor, Department of Community Medicine, D. Y. Patil University, Pune, India

**Keywords:** Ischemic stroke, laboratory, neutrophil, lymphocyte ratio

**TO THE EDITOR**

We would like to share with you our thoughts on “First Admission Neutrophil–Lymphocyte Ratio May Indicate Acute Prognosis of Ischemic Stroke.”^1^

In the conclusion of their abstract, Alpua et al. state that “first admission NLR [neutrophil–lymphocyte ratio] can be used for acute prognosis of ischemic stroke.”^1^ We agree that NLR might be useful. However, it is also necessary to be concerned about quality control and test variability. The neutrophil and lymphocyte values received from different types of automated hematological analyzers may differ,^2^ hence precision analysis of the analyzer being used is necessary. There are also many possible confounding conditions that can result in aberration of either neutrophil or lymphocyte values. This is a main limitation for using NLR as a biomarker in laboratory medicine.^3^

## Abbreviations

NLR: neutrophil-lymphocyte ratio.
